# X-ray spectroscopy station for sample characterization at ELI Beamlines

**DOI:** 10.1038/s41598-023-43924-y

**Published:** 2023-10-12

**Authors:** A. Zymaková, M. Precek, A. Picchiotti, W. Błachucki, I. Zymak, J. Szlachetko, G. Vankó, Z. Németh, J. Sá, T. Wiste, J. Andreasson

**Affiliations:** 1 ELI Beamlines Facility, The Extreme Light Infrastructure ERIC, Za Radnicí 835, 25241 Dolní Břežany, Czech Republic; 2 Hamburg University and The Hamburg Centre for Ultrafast Imaging, Luruper Chaussee 149, 22761 Hamburg, Germany; 3 Institute of Nuclear Physics PAN, Radzikowskiego 152, 31-342 Kraków, Poland; 4 National Synchrotron Radiation Centre SOLARIS, Czerwone Maki 98, 30–392 Kraków, Poland; 5 Wigner Research Centre for Physics, Konkoly-Thege Miklós 29-33, Budapest, 1121 Hungary; 6 Uppsala University, Lägerhyddsvägen 1, SE-751 05 Uppsala, Sweden

**Keywords:** Materials science, Physics

## Abstract

X-ray spectroscopy is a demanded tool across multiple user communities. Here we report on a new station for X-ray emission spectroscopy at the Extreme Light Infrastructure Beamlines Facility. The instrument utilizes the von Hamos geometry and works with a number of different sample types, notably including liquid systems. We demonstrate a simple and reliable method for source position control using two cameras. This approach addresses energy calibration dependence on sample position, which is a characteristic source of measurement uncertainty for wavelength dispersive spectrometers in XES arrangement. We also present a straightforward procedure for energy calibration of liquid and powder samples to a thin film reference. The developed instrumentation enabled us to perform the first experimental determination of the Kα lines of liquidized K_3_Fe(CN)_6_ as well as powdered and liquidized FeNH_4_(SO_4_)_2_. Finally, we report on proof-of-principle use of a colliding jet liquid sample delivery system in an XES experiment.

## Introduction

The advent of large-scale user facilities capable of providing high-energy photon beams from accelerated electrons of high-flux (synchrotrons) or high peak brilliance (X-ray Free Electron Lasers), has provided scientists with enticing capabilities complementary to those of in-house laboratory equipment^1^. In addition, laser-driven sources offer pulsed X-rays with characteristics relevant for X-ray spectroscopy applications e.g., in the form of plasma X-ray^2–4^ and Betatron radiation^5^ sources.

The Extreme Light Infrastructure (ELI) Beamlines Facility is a recently built user-oriented facility where cutting-edge high intensity short-pulse lasers (e.g.^6^) are used for a wide range of applications, including generation of X-rays and their use in spectroscopy applications. For applications in Condensed Matter Science (to study electron and structural dynamics in thin film, powder, and liquid phase samples), a first generation modular X-ray spectroscopy station has been deployed together with a Water-Jet Plasma X-ray Source driven by a high repetition rate (1 kHz) laser^7^. An upcoming spectroscopy station that uses Betatron radiation^8^ is also being designed. For both stations, a modular design permits either absorption or emission spectroscopy, depending on the details of the user case, as well as convenient adaptation to an X-ray source geometry.

Due to the limited availability of beamtime at user facilities in general and ultrashort pulsed X-ray sources in particular, as well as technical demands associated with time-resolved X-ray experiments, the need for sample pre-characterization with conventional laboratory sources remains profoundly relevant^9^. Furthermore, to be as reliable and relevant as possible, this pre-characterization is ideally done in a setup that, to the greatest extent possible, matches conditions of the subsequent time-resolved X-ray experiments, including similar instrument geometry, sample refreshment systems, detectors etc.

This work reports on the characteristics and capabilities of a newly established station for steady-state X-ray spectroscopy developed to facilitate steady-state research^10^, as well as sample pre-characterization for time-resolved experiments at the laser driven pulsed X-ray sources of the ELI Beamlines facility.

Baseline performance as well as an ability to reliably switch between different types of samples are demonstrated. Fe-based samples are studied in foil, powder and liquid forms, with Kα_1_ and Kα_2_ features measured in a sequence of X-ray emission spectroscopy (XES) experiments. For cases involving different sample types, positioning reliability for calibration is examined in a three-step procedure:

1. A solid (foil) standard is measured and the absolute energy scale for the present experiment is derived, knowing the Kα_1_ and Kα_2_ energies from the literature^11^.
2. A powder sample of K_3_Fe(CN)_6_ is positioned, using a new method based on a set of microcameras, so that the center of thickness of the powder sample coincides within 30 µm to that of the solid standard. The Kα_1_ and Kα_2_ emission from the powder sample is measured and the energy scale defined from the measurement of the foil standard is used to calculate the center of gravity (COG) of K_3_Fe(CN)_6_ powder XES spectrum.
3. The presently obtained value matches well with the value of the Kα emission COG for powder K_3_Fe(CN)_6_ available in the literature^12^ verifying the reliability of our sample positioning method when going between sample types.

Using the same camera-based method for sample positioning, we investigate for the first time the XES spectra of K_3_Fe(CN)_6_ and FeNH_4_(SO_4_)_2_ powders in liquid solution using a Wire Guided Jet (WGJ) sample delivery system. Our results verify that the system can perform reliable XES measurements of solid, powder and liquid samples.

## Methods

### Layout and instrumentation: X-ray spectrometer for steady-state XES reference measurements

In an XES experiment, the photon energy distribution of the fluorescence signal created on the target atoms is analyzed. To achieve this, different approaches can be used. One can utilize a detector that immediately extracts/determines the energy of an arriving photon, such as a transition-edge sensor (TES)^13^. Such detectors, however, offer limited energy resolution. Alternatively, dispersive crystals can be employed^14–16^. Such a solution provides higher energy resolution at the cost of losing photons due to the reflective geometry of the instrument.

**Figure 1:**
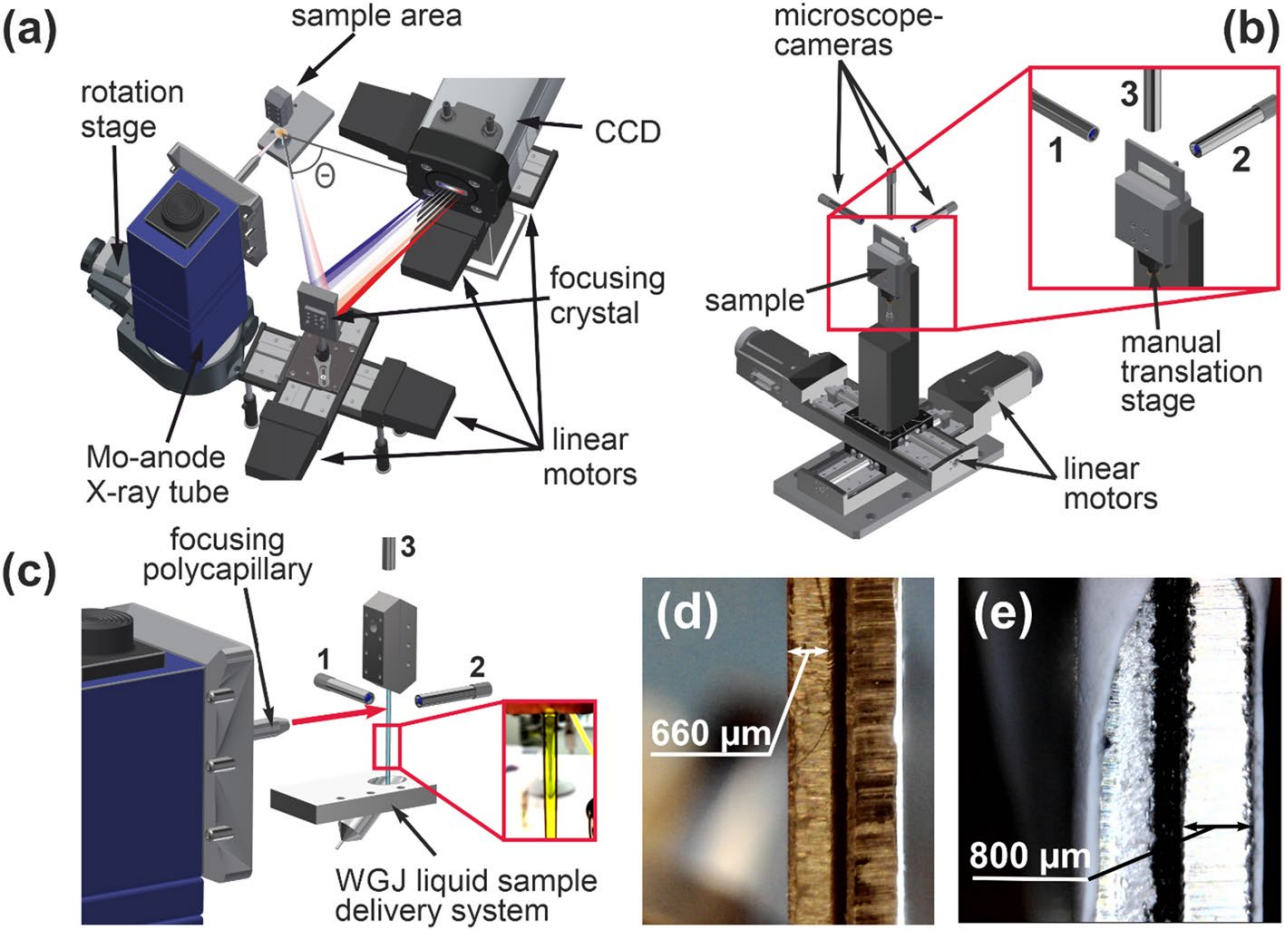
(**a**) Layout of the von Hamos XES end station using the CW Mo source (offline mode). Sample fluorescence is shown in orange. (**b**) Sample tower for thin films and powder samples with 3-camera position control/monitoring system. (**c**) Liquid sample delivery system with numbers designating cameras for sample position control. Red arrow shows X-ray beam direction. The inset demonstrates recycling of concentrated hexacyanoferrate III liquid in the WGJ system. (**d**) Iron standard and (**e**) powder sample profiles as recorded by microcamera 1.

The ELI Beamlines X-ray spectrometer utilizes the von Hamos geometry^17^, see Fig. 1a. In short, a von Hamos spectrometer is built on an isosceles triangle created by a sample, a focusing crystal and a detector where the energy-dependent angle θ is calculated based on Bragg’s law. The crystal, bent in one dimension, disperses broadband radiation generated in the sample and focuses it on a 1D or 2D position-resolving/sensitive detector. This geometry was chosen as the starting geometry for ELI Beamlines X-ray spectroscopy development due to its compactness, versatility and relative ease of use. Expected laser driven radiation sources for time-resolved X-ray spectroscopy applications at the ELI Beamlines facility include Plasma X-ray Sources (as introduced in^7,10,18^) and Betatron radiation sources^19^, for which the von Hamos geometry can be adapted. In addition, as we show in the present publication, the von Hamos geometry can be used to obtain high quality steady-state data. Finally, due to its compactness, it is a suitable geometry where space is limited, e.g. in confined radiation shielded areas or inside vacuum/gas purged chambers.

For the present steady-state experiment the von Hamos spectrometer is combined with a compact air-cooled Molybdenum-anode X-ray tube from XOS with a maximum power of 50 W fixed to a 360° rotation stage for X-ray beam direction specification. The X-ray tube is equipped with a polycapillary focusing optic^10,18^ that has a 30 mm focal length and 133.1 µm spot diameter in the focal plane (FWHM @ 6 keV).

This continuous wave (CW) X-ray source has a fixed location in the spectrometer design, while the dispersive crystal and the detector are mounted on two perpendicular linear motorized stages each. The crystal, of 100 × 50 mm^2^ size, is made from a Si wafer glued to an aluminum substrate with a 250 mm bending radius. The crystal-mount is equipped with piezoelectric motors for remote fine alignment. Currently, crystals with (111), (110) and (100) orientations are available. These cuts allow for X-ray diffraction in (333), (444), (666), (440), (880), (400), (800) orders that continuously cover an X-ray energy range from 5 to 15 keV when operating the spectrometer in the 40° to 73° Bragg angle range. Additional (531) and (553) cuts are planned for spectroscopic experiments exploring operation at high Bragg angles.

As an X-ray detector, a customized charge-coupled device (CCD) detector (Andor, Newton) is used. The detector has a front-illuminated deep-depleted sensor (26.6 × 6.6 mm^2^ chip size) that provides improved handling of charge re-distribution between neighboring pixels. For experiments with polychromatic sources and continuous radiation it is important to be able to do energy-dependent analysis with the CCD detector to allow efficient examination at different diffraction orders given by the crystals. Due to the triangular experimental geometry, the photon beam hits the detector at an angle. In our customized camera, the chip is significantly elevated and mounted closer to the faceplate. This design results in a blind angle of only 39°. For comparison, in a standard camera the sensor is mounted deeper into the housing, limiting the acceptance angle at the high Bragg angle side to 73.7°. This customization allows a much more efficient use of the sensor area, increasing the spectral energy range captured by the detector in each measurement. It can be noted that a photon counting Eiger X 1M detector (Dectris) is available as an alternative to the X-ray CCD.

The spectrometer has been previously tested with one of the ELI Beamlines laser-driven X-ray sources, namely, a water-jet plasma X-ray source. The results have been reported elsewhere^10,18^. For time-resolved experiments there is also a Cu-tape source under commissioning. Our first dosimetry measurements suggest that the total fluxes of our Cu-tape PXS and Mo-anode X-ray tube are comparable.

### Sample environments

The XES end station is equipped to accommodate various forms of samples—solids, powders and liquids (see Fig. 1b,c). All samples are mounted on a perpendicular pair of motorized alignment stages with 0.5 µm positional accuracy. For calibration purposes, a range of standard materials with known reference spectra is available.

Thin film and powder samples can be mounted between two frames in a sandwich structure that allows the middle of the sample thickness to be determined as required for energy calibration (see the next section). The mounting frames for solid thin-film samples and powders have equal length, width and height but vary in terms of the window size (i.e. the dimensions of open sample surface area), see Fig. 1d,e. For film samples, the openings are equal and symmetric. In the powder cage, the front frame facing the source has an equally sized window, but the back frame is largely solid for better support of the sample, with only a small hole (2.7 mm diameter) for X-ray beam transmission, to avoid generation of unwanted noise.

Powder samples are secured between two Kapton sheets, with the Kapton sachet sandwiched between the frames. For XES experiments, the variation in powder thickness within the X-ray focus (~ 100 μm) can be neglected.

For liquid samples in time-resolved experiments, where sample circulation is necessary for each X-ray pulse to expose a fresh sample portion^20^, two liquid-sample delivery systems are prepared for users. The wire-guided jet system (WGJ)^21^ delivers a thin liquid sheet with a thickness in the range of 400 down to 50 µm and requires a relatively small liquid sample volume of only 5–10 mL. The flow rate of the system accommodates an X-ray repetition rate of up to 4 kHz, which is more than sufficient for the available 1 kHz drive lasers, including the in-house developed L1 Allegra^6^. The basic principle of operation behind the WGJ system is surface tension, wherein a liquid flowing between two wires takes the form of a sheet. The liquid is circulated in a closed loop by a microfluidic gear pump and provides a very stable laminar sample sheet. The reproducibility of the liquid sheet position between consequent startups of the pump has been determined to be 43 µm + −5 µm, leading to an experimental error in an XES measurement due to the sample position deviation (for consequent startups of the pump) as low as 0.12 eV. Position reproducibility protocols are available upon request. Further development of the WGJ system is ongoing^22^.

A second type of liquid sample delivery system available for user operation is a colliding jet system^23^ . This system generates a free-flowing flat liquid sheet with a thickness of only a few microns by colliding two liquid flows at the nozzle of a chip with three microfluidic channels, or, alternatively, by compressing the liquid exiting the central channel with gas flows from the outer channels. In order to compensate for solvent evaporation and keep sample concentration constant, the systems incorporate a syringe pump that continuously dilutes the sample medium.

## Results and discussion

### Energy calibration in X-ray emission spectroscopy experiments

**Figure 2:**
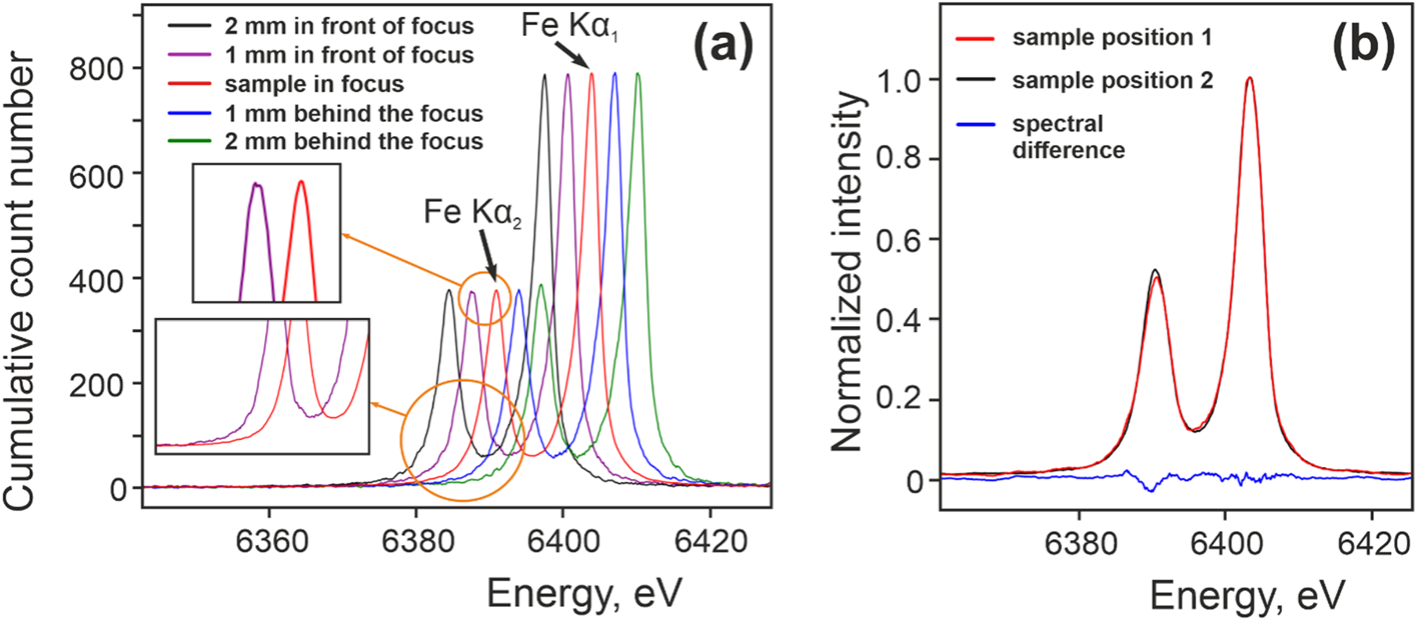
(**a**) Movement of iron Kα_1_ and Kα_2_ peaks due to shifts in the sample position by 1 mm steps with respect to the focal point. The amplitudes of the spectra are normalized to the purple curve. The insets demonstrate the variation in spectral shape depending on the acquired statistics. As a quality measure, the red curve was acquired during 5.5 h (4100 frames), while the purple curve was obtained in 24 min (300 frames). The top inset shows the difference in the quality of the Kα_2_ peak for a measurement with relatively poor (magenta) and good (red) statistics. Sample—iron foil of 7.5 µm thickness. (**b**) Comparison of two amplitude normalized XES spectra acquired from the same sample, removed and returned after two days with the position adjusted to references made on the first day using two microcameras. The blue curve shows the spectral difference between the spectra. The difference in Kα_2_ intensity is attributed to different statistics obtained through different acquisition times.

When working with polychromatic sources like CW X-ray tubes or plasma X-ray sources, calibration becomes particularly complicated in the case of XES. Significant efforts were made to address this issue in the development of the described experimental station. A shift of XES features across the detector area can be caused not only by physical and chemical processes, but also by deviations in the sample position along the X-ray beam. Figure 2a shows a sequence of XES spectra from an iron calibration foil (EXAFS materials) acquired by first placing the sample in focus and subsequently scanning 4 additional sample positions with 1 mm steps. The red curve (spectrum acquired in the focal plane) has been referenced to Kα_1_ and Kα_2_ energies given in^13^. The average shift of the spectral peaks is ~ 3.17 eV per 1 mm, derived at the FWHM of the peaks, corresponding to a step of 28 pixels on the camera chip. This information allows us to determine the energy precision of the spectrometer for the present experimental campaign (i.e. at energies around 6.4 keV) as 0.11 eV/pixel. This value corresponds well with expectations^24^ and allows, for example, the study of chemical shifts of emission lines or the structure of valence-to-core transitions with satisfactory precision^25^.

In a standard XES experiment, the energies of characteristic features of a material under study are compared to reference spectra from a well-known reference sample. In practice this means that a calibration sample has to be measured in sequence with the sample of interest, so that the energy axis can be derived experimentally when using broadband sources. This approach requires a reliable method for referencing the sample position as attempts to ad hoc place a calibration sample (i.e. an iron calibration foil) in the same position as the sample under study, using e.g. a clamping mechanism or fixing screws easily results in deviations of the sample position of the order of 500 μm which, for the present experiment, would introduce an experimental error of 1.59 eV.

Therefore, high precision control of the sample position must be maintained during the necessary sequence of experiments and reference measurements. This becomes particularly challenging when measuring different types of samples, in particular when data from liquid samples need to be calibrated to a thin-film reference. To deal with this challenge we have developed the following procedure: three microscope-cameras were added to the setup and focused on the sample. Transversal cameras 1 and 3 (see Fig. 2b) were focused at the center of the sample sandwich structure (a thin foil between two frames of 660 µm and 800 µm thickness), while coaxial camera 2 had the sample surface in focus. The sample position along the X-ray beam was aligned to the middle of the sample thickness using cameras 1 and 3, and cross-checked using camera 2. The focal depth of the cameras has been experimentally determined to be 10–20 µm. The use of cameras allowed us to achieve a transversal sample placement precision of as fine as 20–30 µm (resulting in an experimental error as low as 0.063 eV). The accuracy of positioning was limited by the topology of the calibration sample holder (see Fig. 1d) and can be further enhanced by polishing the sample frame to improve on the surface quality, or by marking a reference plane, as the cameras are able to resolve sample movements of a few microns. When using the coaxial camera to focus on the sample surface, the variation in thickness of various samples needs to be taken into account. For example, in the experimental campaign described here, the thickness of the Fe foil was 7.5 µm, while the liquid sheet from the WGJ had a thickness of 370 µm. The thickness of the powder sample varied from a minimum of 120 µm, the thickness of two Kapton foils in total, plus the powder thickness on the order of 1 to tens of microns.

It can be noted that only two cameras are strictly necessary to determine the absolute transversal sample position along the X-ray beam (see Fig. 1c). Depending on details in the present experimental arrangement we chose to use a third camera for verification where the experimental geometry allowed.

We illustrate the reproducibility of the sample positioning using the microscope camera technique in a sequence of XES measurements from an iron standard foil using a Si (333) focusing crystal at a Bragg angle of 67.87°. Figure 1d shows a profile of the calibration iron foil in the mounting frames. A calibration sample was placed in the sample holder, the position of its center plane was assessed and marked as a reference, and a short acquisition of an emission spectrum was done. Then the sample was removed and returned two days later (with a lot of manipulation in the sample area in between). The sample position was adjusted using only two cameras with the result shown in Fig. 2b. The spectral difference (blue curve) shows mainly a statistical discrepancy between the spectra due to different acquisition times of 16 min (black curve) and 104 min (red curve). In this verification, the precision of the optical alignment system gives a ΔCOG = 0.019 eV. We conclude that the method is reliable and can be used for energy calibration in XES measurements.

The utility of the cameras in the system is not limited to sample position control. The coaxial camera also detects/displays the presence of X-rays as a sparkling signal that can be used to determine the position/direction of the X-ray beam by a knife-edge measurement. As the chip of microscope camera 2 located behind the sample can be damaged by high-energy photons, it needs to be protected from X-rays transmitted through the sample. In our setup X-ray radiation is filtered by a set of glass slides (not shown in Figures). The number of slides was chosen to allow a suitable level of radiation to reach the camera.

### X-ray emission spectroscopy on powder samples

**Figure 3:**
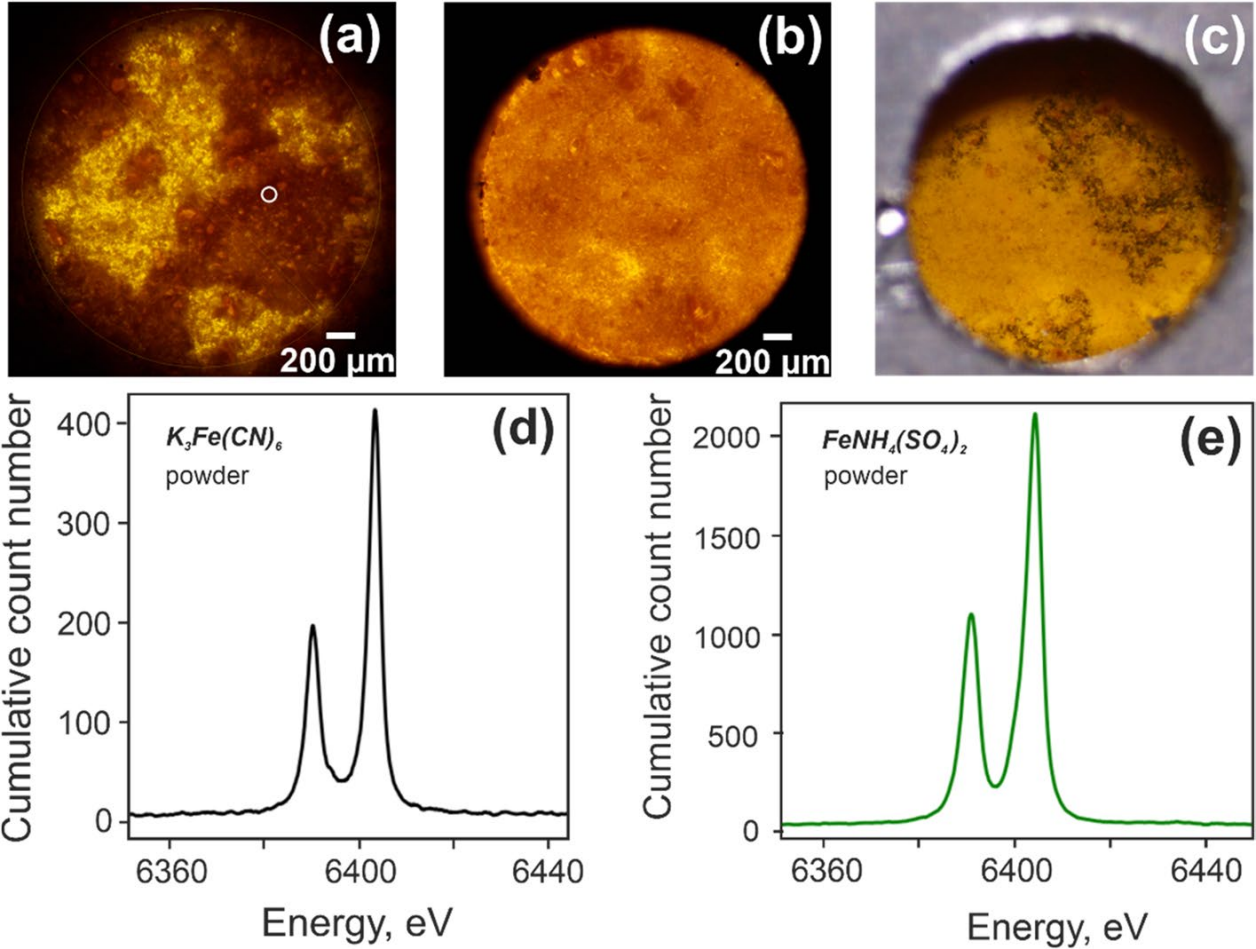
(**a**) Microscopy image of the K_3_Fe(CN)_6_ powder sample in the sample cage with the opening for X-ray transmission. The white circle indicates the X-ray impact area. (**b**) Microscopy image of the FeNH_4_(SO_4_)_2_ powder sample. (**c**) Back side of the sample cage for powders captured by the coaxial camera 2. (**c**) Microscopy image of the FeNH_4_(SO_4_)_2_ powder sample. (**d**) Measured XES spectrum of powder K_3_Fe(CN)_6_. Acquisition time is 6 h. (**f**) XES spectrum of powder FeNH_4_(SO_4_)_2_. Acquisition time is 16 h. COG value of this spectrum is ~ 6399.69 eV.

Experimental results acquired using iron foils are shown in the previous section (Fig. 2). In the next phase of the campaign, two iron-based powders (Fig. 3a–c) were examined—K_3_Fe(CN)_6_ (hexacyanoferrate 3 +) and FeNH_4_(SO_4_)_2_ (iron ammonium sulfate). The first one was ground to a powder and placed between two sheets of Kapton foil. Iron ammonium sulfate was dried in a desiccator for 4 days, after which a similar grinding procedure was applied and the resulting powder was placed between Kapton foils. For the measurements the powder samples were placed between two frames in a sandwich structure, as described in the *Sample environments* section. The results acquired from both powder samples were referenced to the iron calibration foil using the microcamera method. The obtained spectra are shown in Fig. 3d,e. In order to investigate the precision in our measurements, we calculate the COG value of the measured K_3_Fe(CN)_6_ spectrum. The calculated COG is 6399.2609 eV and perfectly matches the value measured at the European Synchrotron Radiation Facility (ESRF), France^12^. Thus, we conclude that energy calibration using the microcamera method delivers reliable results also for powder samples.

The difference between the COGs of the two iron-based powders is as little as 0.43 eV. XES measurements without the microscope-camera calibration method would not allow this shift to be resolved as the value is well below the experimental error of 1.59 eV (see section *Energy calibration in X-ray emission spectroscopy experiments*).

### X-ray emission spectroscopy on liquid samples

For experiments with the WGJ, the position of the vertical guiding wires of the WGJ system was adjusted to that of the calibration foil using the microscope-camera technique ensuring energy calibration of the spectrometer. The design of the WGJ allows samples to be changed/exchanged without touching the guiding wires or moving the sample circulation system. This sample exchange is achieved by detaching a flexible soft pipe, flushing the system, and then filling it again with a new sample liquid in the area of a damper (see^21^). Consequently, the position of the liquid sample remains permanent, eliminating the risk for placement error during sample exchange.

**Figure 4:**
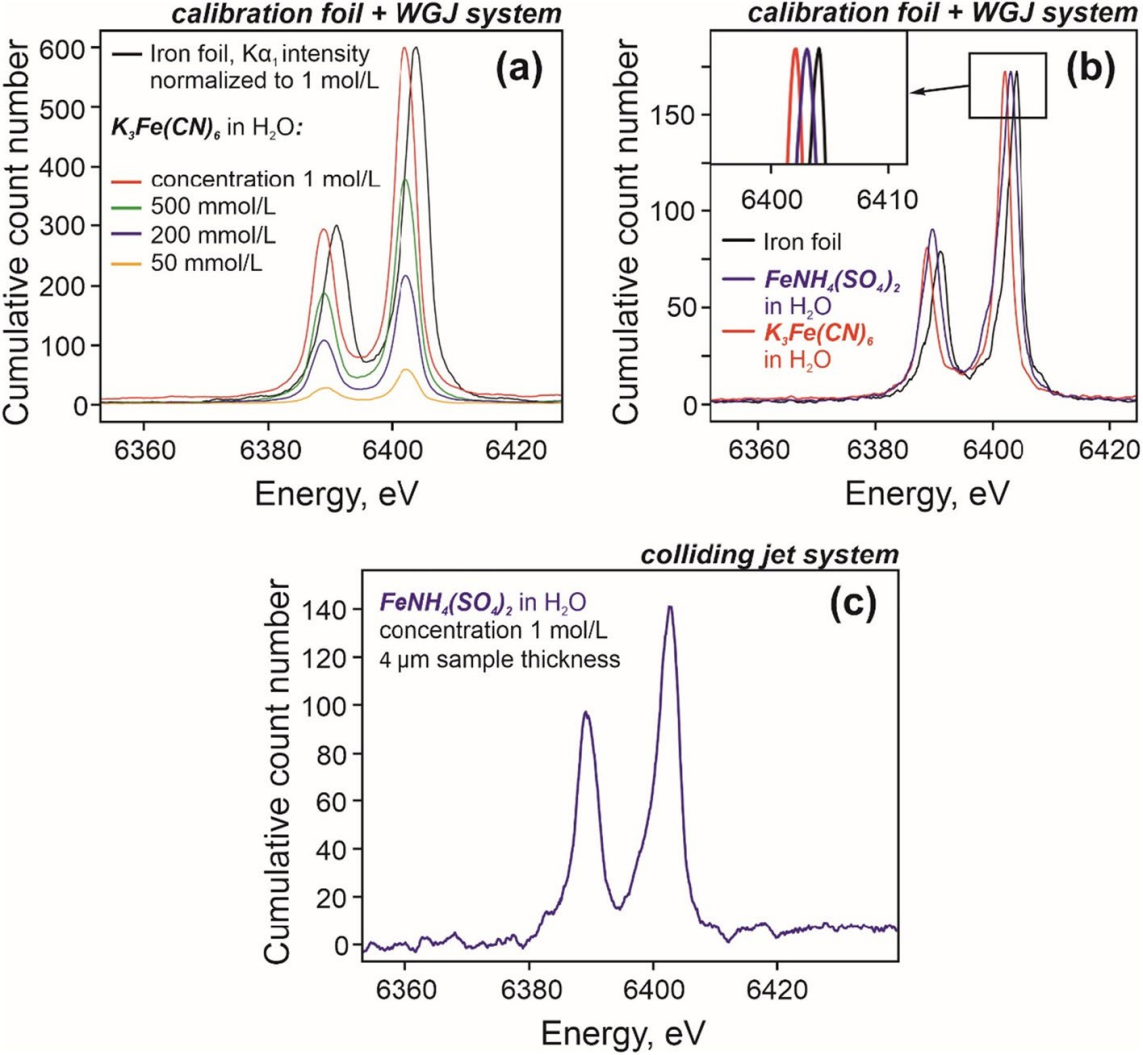
(**a**) XES spectra of hexacyanoferrate III solution in water for different concentrations compared to the XES spectrum of an iron foil shown for energy reference. The solutions were circulated in the WGJ system at a constant flow rate corresponding to a sheet thickness of 370 µm. Acquisition time of each spectrum from the liquid sample is 2 h. (**b**) Chemical shifts of 1 mol/L solutions with the energy and Kα_1_ peak amplitude calibrated to the iron foil standard. (**c**) XES spectrum of FeNH_4_(SO_4_)_2_ water solution delivered by the colliding jet liquid sample delivery system, baseline subtracted, corrected for a spatial shift of 500 µm (a result of sample positioning using only one microcamera).

A set of data with different sample concentrations was collected in order to compare the energies of the characteristic features and understand the impact of the concentration on the acquisition time required for the accumulation of sufficient statistics (see Fig. 4). The presented data have been acquired using the WGJ with pulsations dampener installed.

Both liquids show a lower energy for the characteristic lines compared to pure Iron, and the characteristic energies from aqueous K_3_Fe(CN)_6_ are lower than those from FeNH_4_(SO_4_)_2_. This result correlates well with the data obtained from powders (Fig. 3d,e). As expected, the intensities of the Kα_1_ and Kα_2_ characteristic features decrease when the concentration is reduced due to a smaller number of iron atoms per irradiated sample volume in the diluted samples.

After establishing operations with the WGJ system we performed a proof-of-principle XES measurement using a colliding micro-liquid jet system. The system delivers a very fine liquid sheet (a few microns thick)^23^, and long acquisition times are required as the collected statistics per time unit becomes low. Experiment data, shown in Fig. 4c, were collected over more than 16 h. Nevertheless, clear XES features have been successfully detected. Certainly, the time required for collecting reasonable statistics with the present colliding jet liquid sample delivery system in combination with the used X-ray source is impractically long, due to a small cross section of X-rays with the liquid sample, and a relatively small thickness of the colliding jet. However, the system does offer advantages such as comparably low evaporation rate and higher pump reliability. As such, it may be an option for specialized scientific projects and at X-ray sources of much higher brilliance, e.g. at synchrotrons. Development of the colliding jet system that can provide liquid samples with a sheet thickness of 100–200 μm is ongoing. Such sample thickness would allow us to acquire decent statistics in a more manageable time frame.

The advantage of a microfluidic nozzle system, compared to the WGJ, is the absence of the freestanding guiding wires. This is expected to provide higher reproducibility for the liquid sheet position and remove a potential source of secondary emission from the vicinity of the examined spot. The system also delivers much flatter sample sheets, compared to the WGJ, due to the completely different microfluidic regime of the two systems. Thus, it has great potential for IR pump-X-ray probe experiments (especially in XAS geometry).

## Conclusions and outlook

This work demonstrates a capacity to perform detailed and novel steady-state XES experiments using a CW version of the ELI Beamlines time-resolved X-ray spectroscopy station setup. We verify its ability to investigate thin film, powder and liquid samples. High level of similarity between the steady-state system described here, and the time-resolved X-ray spectroscopy^7^ setup under development means users will be able to prepare for more complex time-resolved experiments with minimal consumption of available beamtime. In particular, for liquid samples, critical parameters like preferred jet/sheet generation method, sample concentration, solvent evaporation rate, etc. can be optimized before work starts on the time-resolved setup. In addition, the CW station can serve as a testing ground for researchers focusing on the synthesis of new materials and compounds who do not have access X-ray spectroscopy capabilities in their home laboratories. This can be particularly useful for the users of the ELI Beamlines stations for ultrafast optical spectroscopy who may be interested in complementary steady-state or time-resolved X-ray data^26,27^.

A robust approach to control and reference the sample position using a set of microcameras has also been described. This method allows us to improve on the reproducibility of the sample position by a factor of about 25, reducing systematic errors in energy calibration that may otherwise alter XES experiment outcomes by obscuring spectral shifts related to physical and chemical processes in a sample. Verification data are collected from different forms of Iron-based samples—solid, powders and liquids. We assume that alignment of the thickness central axis of samples in different physical forms eliminates the experimental errors given by the position shift as much as possible. However, for the most reliable result we suggest comparing samples of the same physical form and thickness.

Our results show the capability of the newly established station to detect shifts of the COG of K-fluorescence features of the order on ~ 0.019 eV (i.e. well below the lifetime broadening of the 1s shell)^28^. This precision is sufficient for high sensitivity studies of the electronic structure around the photo-excited atoms. X-ray beam divergence of the current setup has been measured to be 3.8°^29^. If desired, the resolution of the setup can presumably be improved by decreasing the beam divergence by means of a Soller slit^30^ at the cost of a decrease of the photon flux on the sample.

We note that the sensitivity of XES to the sample position (see Fig. 2a) can also be addressed using a dispersive refocusing geometry. This approach implies placing the X-ray point source off the spectrometer equator^14,15^. We do not rule out the possibility of applying this approach. However, we chose to implement the camera-based referencing system in the initial realization of the spectrometer setup for steady state and time-resolved X-ray spectroscopy at the ELI Beamlines facility.

Ongoing upgrades to the setup include increasing the degree of motorization and automation of the system for faster and more precise remote alignment and control. Development of the sample environment is ongoing as well. Planned improvements include motorization of the vertical translation of the platform for the liquid jet sample delivery system. For liquid sample delivery systems with a non-uniform thickness in the vertical direction (e.g. WGJ and colliding jet) such vertical movement grants a possibility to select the sample thickness. This provides added value to the setup for optical pump-X-ray probe experiments.

Longer term developments include an upgrade to a multi-crystal detector that will enhance spectrometer efficiency through faster acquisition of statistics. A multi-crystal detector with 9 mirror elements that can be individually aligned by piezo actuators forming a common 250 mm radius cylindrical surface is in the design phase. We also plan to upgrade the spectroscopy station by an implementation of parallel emission and absorption setups. With this upgrade, radiation generated by a single X-ray source on one sample can be simultaneously gathered in XAS and XES geometries^31^, allowing parallel studies of occupied and unoccupied electronic states.

The present verification of an XES station for steady-state measurements represents an important step towards an ELI Beamlines facility goal of offering a versatile station for time-resolved X-ray spectroscopy, complemented by a satellite station for steady-state measurements for sample characterization and methods development to an international user community.

## Data Availability

The authors confirm that the data supporting the findings shown in this paper are available within this article. Raw data that support the findings of this study are available from the corresponding author, upon request.
